# Challenging Occam’s Razor: Dual Molecular Diagnoses Explain Entangled Clinical Pictures

**DOI:** 10.3390/genes13112023

**Published:** 2022-11-03

**Authors:** Beatrice Spedicati, Anna Morgan, Giulia Pianigiani, Luciana Musante, Elisa Rubinato, Aurora Santin, Giuseppe Giovanni Nardone, Flavio Faletra, Giorgia Girotto

**Affiliations:** 1 Department of Medicine, Surgery and Health Sciences, University of Trieste, 34149 Trieste, Italy; 2 Institute for Maternal and Child Health-I.R.C.C.S. “Burlo Garofolo”, 34137 Trieste, Italy

**Keywords:** dual molecular diagnosis, multilocus genomic variation, whole exome sequencing

## Abstract

Dual molecular diagnoses are defined as the presence of pathogenic variants at two distinct and independently segregating loci that cause two different Mendelian conditions. In this study, we report the identification of double genetic disorders in a series of patients with complex clinical features. In the last 24 months, 342 syndromic patients have been recruited and clinically characterised. Whole Exome Sequencing analysis has been performed on the proband and on both parents and identified seven patients affected by a dual molecular diagnosis. Upon a detailed evaluation of both their clinical and molecular features, subjects are able to be divided into two groups: (A) five patients who present distinct phenotypes, due to each of the two different underlying genetic diseases; (B) two patients with overlapping clinical features that may be underpinned by both the identified genetic variations. Notably, only in one case a multilocus genomic variation was already suspected during the clinical evaluation. Overall, our findings highlight how dual molecular diagnoses represent a challenging model of complex inheritance that should always be considered whenever a patient shows atypical clinical features. Indeed, an accurate genetic characterisation is of the utmost importance to provide patients with a personalised and safe clinical management.

## 1. Introduction

The ultimate goal of Medical Genetics is the identification of the molecular cause of genetic disorders. These conditions, albeit singularly rare, present a population prevalence of 3.5–5.9% [1], thus posing a major burden from a medical, social, and economical point of view. In this light, achieving a correct molecular diagnosis has a positive impact not only on patients and their families but also on the healthcare system. In fact, understanding the genetic basis of a given disorder could implement specific indications for correct follow-up and potential treatment, avoid ineffective medical care, provide prognosis information, and identify familial recurrence risk.

Usually, medical geneticists hypothesise clinical diagnoses of Mendelian diseases by recognising peculiar syndromic patterns. As a consequence, whenever signs and symptoms do not precisely fit into a known model, either an apparently new condition or a phenotypic expansion of a single disorder are usually considered [2]. However, the introduction of high-throughput sequencing technologies, such as Whole Exome Sequencing (WES), that allow the simultaneous analysis of thousands of genes, have highlighted another fascinating possibility: the coexistence of dual molecular diagnoses [2,3,4]. These are defined as the presence of pathogenic variants at two distinct and independently segregating loci that cause two different Mendelian conditions [3]. Since the introduction of WES in the clinical setting, several works have reported the identification of subjects affected by dual molecular diagnoses, with an overall rate of 5–7% [2,3,4].

In consideration of the growing number of reports of double or even triple genetic diagnoses [5,6,7,8,9,10,11], increasing attention must be paid to entangled clinical pictures, and atypical features should prompt awareness of possible multiple causes. This is of the utmost importance to provide patients with the best and safest standards of care, allowing for a more personalised and appropriate clinical management [7].

To further investigate the peculiar scenario of multilocus genomic variation, here we present the identification of patients affected by dual molecular diagnoses, within a cohort of 342 patients who underwent WES analysis in the last 24 months.

## 2. Materials and Methods

### 2.1. Ethical Statement

All the analyses have been performed following relevant guidelines and regulations. Written informed consent was obtained from all participants or their legal guardians. The study was conducted in accordance with the tenets of the Helsinki Declaration and was approved by the Ethics Committee of the I.R.C.C.S. “Burlo Garofolo” of Trieste.

### 2.2. Clinical Evaluation

In the last 24 months, 342 unrelated patients with complex clinical pictures and suspicion of underlying genetic disorders have been referred to the Medical Genetics Unit of the I.R.C.C.S. “Burlo Garofolo” (Trieste, Italy).

All participants were characterised through a detailed anamnesis, a dysmorphological examination and further investigations. A familial anamnesis has been collected in order to identify possible other affected family members and a personal medical history has been obtained to highlight potential confounding factors (i.e., infections, trauma, or other non-genetic causes of congenital abnormalities). A physical examination has been carried out to identify dysmorphic features, with particular attention to facial, ectodermal, skeletal, and genital features. Ancillary clinical tests have been performed when appropriate and included electroencephalogram (EEG), brain Magnetic Resonance Imaging (MRI) and Computed Tomography (CT), electrocardiogram (ECG) and echocardiography, abdominal ultrasound, neurological, ophthalmological, otorhinolaryngoiatric, and cardiological evaluations.

### 2.3. DNA Extraction and Quality Control

Genomic DNA was extracted from peripheral whole blood samples of patients and, whenever available, both their parents using the QIAsymphony^®^ SP instrument with QIAsymphony^®^ Midi Kit (Qiagen, Venlo, The Netherlands), following manufacturer’s instructions. DNA quality was verified with 1% agarose gel electrophoresis and its concentration was measured using Nanodrop ND 1000 spectrophotometer (NanoDrop Technologies Inc., Wilmington, DE, USA).

### 2.4. WES and Data Analysis

WES was performed on an Illumina NextSeq 550 instrument (Illumina Inc., San Diego, CA, USA) using the Twist Human Core Exome and Human RefSeq Panel kit (Twist Bioscience, South San Francisco, CA, USA), according to the manufacturer’s protocol. Briefly, genomic DNA was enzymatically fragmented, ligated to a universal adapter and amplified using the Unique Dual Index primers (Twist Bioscience, South San Francisco, CA, USA). Samples were therefore hybridised with the Twist Human Core Exome and the Human RefSeq Panel kit, which cover 99% of protein coding genes. Hybridised fragments have been captured, amplified, and sequenced. Sequencing coverage statistics for all samples and, in detail, for cases affected by dual molecular diagnoses are reported in Table S1.

The process allows the production of FASTQ files that were analysed through a custom pipeline (Germline-Pipeline), developed by enGenome s.r.l. (https://www.engenome.com/) (Figure S1). This pipeline permits the identification of germline variants, including Single Nucleotide Variants (SNVs), short Insertions/Deletions (INDELs), and exon-level Copy Number Variations (CNVs) starting from sequence reads. The secondary analysis leads to the generation of final VCF files that contains SNVs, INDELs and CNVs.

VCF files were analysed through the enGenome Expert Variant Interpreter (eVai) software (https://evai.engenome.com/), that allows variant annotation, interpretation, and prioritisation, exploiting both artificial intelligence and the American College of Medical Genetics and Genomics/Association for Molecular Pathology (ACMG/AMP) guidelines to analyse and classify genomic variants [12].

Variant frequency was verified both in NCBI dbSNP build 155 (https://www.ncbi.nlm.nih.gov/snp/) and gnomAD (https://gnomad.broadinstitute.org/) to exclude variants previously reported as polymorphisms. Pathogenicity of already-reported variants was assessed through The Human Gene Mutation Database (https://www.hgmd.cf.ac.uk/ac/index.php) and ClinVar (https://www.ncbi.nlm.nih.gov/clinvar/). All databases have been accessed lastly on 9 October 2022. The effect of all identified variants was evaluated through several in silico prediction tools, as PolyPhen-2 [13], Sorting Intolerant From Tolerant (SIFT) [14], Pseudo Amino Acid Protein Intolerance Variant Predictor (PaPI score) [15], Deep Neural Network Variant Predictor (DANN score) [16], and dbscSNV score [17]. SNVs leading to synonymous amino acid substitutions not predicted as damaging, not affecting splicing or highly conserved residues were excluded; furthermore, variants with a quality score (QUAL) < 20 or called in off-target regions were excluded as well (Table S2).

Variants were discussed within a multidisciplinary team to verify whether they could be possibly matched to each patient’s phenotypic characteristics; finally, all variants of interest were confirmed by Sanger sequencing. Familial segregation was also performed by Sanger sequencing.

### 2.5. Protein-Protein Interaction Analysis

The Ingenuity Pathway Analysis (IPA) system (version 81348237, Ingenuity Systems; QIAGEN Inc., Hilden, Germany-https://digitalinsights.qiagen.com/IPA, (accessed on 21 October 2022)) was used to identify relationships between genes of interest based on associated functions and data mining from experimental studies reported in the literature. In particular, My Pathways-Build-PathExplorer tool was used for the protein-protein interaction analysis, taking into account only direct interactions and excluding non-coding genes from the analysis.

## 3. Results

Three hundred and forty-two unrelated patients underwent WES analysis in the last 24 months and a molecular diagnosis has been achieved for 138 of them (40.4%). Seven patients presented a dual molecular diagnosis (5.1% of all solved cases), and their clinical and molecular characteristics are detailed in the following paragraphs.

**Figure 1:**
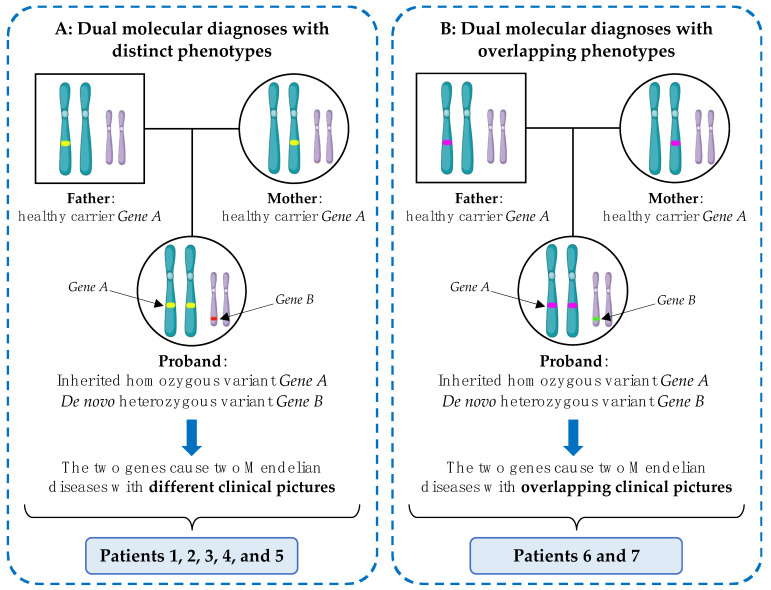
Dual molecular diagnoses patterns. (**A**). Dual molecular diagnoses with distinct phenotypes: patients present a blended phenotype due to the simultaneous presence of two Mendelian disorders caused by variants at two independently segregating loci. Each condition is characterised by a different and specific set of signs and symptoms, and they may be recognised as discrete upon reverse phenotyping. (**B**). Dual molecular diagnoses with overlapping phenotypes: patients’ clinical picture may be ascribed to either of the two underlying Mendelian disorders. In the exemplified cases, one condition is inherited from both patients in an autosomal recessive manner, and the other has a *de novo* origin in the proband; nevertheless, all inheritance patterns are possible.

After a careful examination of both their clinical and molecular features, patients affected by a dual molecular diagnosis may be divided into two different groups (Figure 1, Table 1):

A. Patients who present distinct phenotypes, due to each of the two different underlying genetic diseases. Five patients belong to this category (i.e., Patients 1, 2, 3, 4 and 5).
B. Patients with overlapping clinical features that may be underpinned by both the identified genetic variations. Two patients belong to this group (i.e., Patients 6 and 7).

### 3.1. Patients Who Present Distinct Phenotypes Due to Two Different Underlying Genetic Diseases

#### 3.1.1. Patient 1: Syndromic Craniosynostosis and Xerosis Cutis

**Figure 2:**
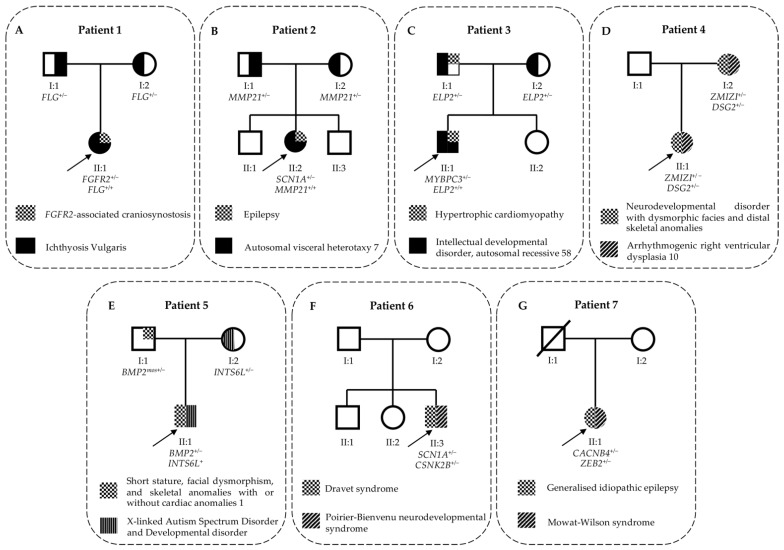
Family pedigrees of patients affected by dual molecular diagnoses. The figure illustrates the main clinical features of each patient and the genes associated with their phenotypes. (**A**–**G**) indicate each of the seven patients described in this study. Half black filling of father and mother indicates healthy carrier parents. Three-quarters black filling of proband indicates that he/she is affected by an autosomal recessive condition. One-quarter or half chessboard filling of proband or parent indicates that subject is affected by an autosomal dominant disorder. Half diagonal-line filling of proband or parent indicates that subject is affected by another autosomal dominant disorder. Half vertical-line filling of a male proband indicates that he is affected by an X-linked disorder. Half vertical-line filling of a female subject indicates a healthy carrier of an X-linked condition.

Patient 1 (Figure 2A) is a 20-day-old girl born after an apparently uneventful pregnancy.

At birth, facial asymmetry, hands abnormalities (i.e., short and enlarged thumbs bilaterally and partial duplication of the distal phalanx of the fifth finger), and feet syndactyly were identified, thus prompting a genetic evaluation. The clinical examination highlighted marked facial and cranial asymmetry, anteverted nares, low-set ears, exaggerated Cupid’s bow, downturned oral commissures, and pointed chin. Furthermore, the newborn showed extensive *xerosis cutis* with multiple desquamative areas. A head CT scan highlighted a complex craniosynostosis with ossification of the right coronal hemi-suture and of both parietotemporal sutures; accordingly, a marked asymmetry of cerebral hemispheres volume and right eye proptosis were identified.

WES was carried out on the proband’s and both her parents’ DNA and data analysis highlighted the presence in the girl of a missense heterozygous variant in the *FGFR2* gene (NM_000141.4) (c.940G>T, p.(Ala314Ser)). Pathogenic variants in *FGFR2* are associated with several autosomal dominant diseases characterised by overlapping clinical features, as Apert syndrome (MIM: #101200), Crouzon syndrome (MIM: #123500) and Saethre-Chotzen syndrome (MIM: #101400), that are collectively known as *FGFR2*-associated craniosynostoses. The identified variant has already been described as pathogenic [18] and familial segregation confirmed its *de novo* origin in the proband.

Furthermore, we identified two compound heterozygous nonsense variants in the *FLG* gene (NM_002016.1). Pathogenic variants in *FLG* cause Ichthyosis Vulgaris (MIM: #146700), both autosomal dominant and autosomal recessive. Both variants (c.7339C>T, p.(Arg2447*) and c.3191G>A, p.(Trp1064*)) have already been reported in association with this phenotype [19,20].

#### 3.1.2. Patient 2: Epilepsy and Congenital Heart Disease

Patient 2 (Figure 2B) is an eight-year-old girl that came to the geneticist’s attention due to congenital heart malformation (i.e., levo-transposition of the great arteries and pulmonary valve stenosis identified at five months of age), drug-resistant epilepsy (i.e., tonic-clonic seizures, firstly appeared at four months of age), and intellectual disability. Her family history is unremarkable, with the exception of the maternal grandmother who is reported to having experienced tonic-clonic seizures from the age of seven to the age of 14.

WES was carried out on the family trio and revealed the presence of two novel likely pathogenic variants in two different genes. The first involves the *SCN1A* gene, known to be associated with several autosomal dominant epileptic disorders (e.g., Dravet syndrome–MIM: #607208–, Developmental and epileptic encephalopathy 6b–MIM: #619317–, and Generalized epilepsy with febrile seizures plus, type 2–MIM: #604403). The second involves *MMP21*, whose biallelic mutations cause Autosomal visceral heterotaxy 7 (MIM: #616749), that includes the transposition of the great arteries.

As regards *SCN1A* (NM_001165963.1), the c.2591_2593delTGC, p.(Leu864del) heterozygous inframe deletion is predicted as damaging by the in silico tool PaPI score, is not reported in any public database, and familial segregation confirmed its *de novo* origin in the proband.

As regards *MMP21* (NM_147191.1), the c.903G>A, p.(Met301Ile) homozygous missense variant affects a highly conserved residue, is predicted as damaging by all in silico tools employed in the analysis, and is reported with a very low frequency in the gnomAD database (Minor Allele Frequency (MAF): 0.0016%). Familial segregation confirmed that both parents are heterozygous carriers and, as expected, do not present any clinical features associated with Autosomal visceral heterotaxy 7.

#### 3.1.3. Patient 3: Syndromic Intellectual Disability and Hypertrophic Cardiomyopathy

Patient 3 (Figure 2C) is an 18-year-old boy affected by severe intellectual disability, spastic tetraplegia, and non-progressive hypertrophic cardiomyopathy. The boy presents severe neurodevelopmental delay: he acquired the sitting position at three years of age, started to walk at four years old and lost this ability at 12 years of age, and never learnt to talk. Furthermore, the proband is affected by conductive hearing loss, bilateral strabismus, and bilateral hip dysplasia. From a phenotypical point of view, he displays deeply set eyes, thick upper lip vermillion, widely spaced teeth, macroglossia, and mandibular prognathism.

As a single-gene defect underlying all his clinical features was suspected, in trio WES was performed. However, data analysis highlighted the presence of two pathogenic variants in two distinct genes. In particular, the boy carries a homozygous missense variant already described as causative of Intellectual developmental disorder, autosomal recessive 58 (MIM: #617270) in the *ELP2* gene (NM_001242875.1) (c.1580G>A, p.(Arg527Gln)) [21], and a heterozygous frameshift variant inherited from the father and already reported in association with Hypertrophic cardiomyopathy (MIM: #115197) in *MYBPC3* (NM_000256.3) (c.913_914delTT, p.(Phe305Profs*27)) [22]. Upon the genetic finding, the father underwent a detailed cardiological examination that did not reveal the presence of a hypertrophic cardiomyopathy, possibly in accordance with the incomplete penetrance and variable expressivity that characterise this disorder.

#### 3.1.4. Patient 4: Syndromic Intellectual Disability and Congenital Heart Disease

Patient 4 (Figure 2D) is 37-year-old woman who sought genetic counselling because of a complex congenital heart malformation and intellectual disability. Her heart malformation had already been detected in the prenatal period and was characterised by interatrial and interventricular defects together with tricuspid valve atresia. In addition, she presented an arrhythmic phenotype, with atrial tachycardia as its main feature. Furthermore, the proband is affected by mild intellectual disability, submucosal cleft palate, moderate bilateral sensorineural hearing loss, and mild facial dysmorphisms (i.e., synophrys, thick upper and lower vermillion, and right earlobe malformation). Concerning the woman’s family history, the mother is also reported to present mild intellectual disability and the same minor facial features.

WES of the family trio revealed the presence in both the proband and the mother of two novel likely pathogenic variants at the heterozygous state, one in the *ZMIZ1* gene (NM_020338.3) and the other in the *DSG2* gene (NM_001943.3). Variants in *ZMIZ1* have been associated with autosomal dominant Neurodevelopmental disorder with dysmorphic facies and distal skeletal anomalies (MIM: #618659), which partially overlaps the clinical features of the proband. The identified missense variant (c.1984A>G, p.(Asn662Asp)) affects a highly conserved residue, is predicted as damaging by the in silico tools used during the analysis, and is not reported any public database. As regards *DSG2*, variants in this gene are known to cause autosomal dominant Arrhythmogenic right ventricular dysplasia 10 (MIM: #610193). WES data analysis showed the presence of a nonsense variant, c.621_626delTCCTCC, p.(Tyr207_Pro209delinsTer), predicted as damaging by the in silico tool PaPI score and already reported as pathogenic in ClinVar (RCV000544613). In consideration of this finding, a specific cardiological follow-up has been performed also in the proband’s mother: an ECG, echocardiogram, and cardiac MRI have been performed and resulted all normal. This may reflect the incomplete and age-dependent penetrance of this disorder, that is estimated to be around 30–50% [23].

#### 3.1.5. Patient 5: Multiple Congenital Malformations and Autism Spectrum Disorder

Patient 5 (Figure 2E) is a four-year-old boy who came to the geneticist’s attention due to the presence of anal atresia, identified at birth and surgically treated, lumbosacral transitional vertebra, recognised in the neonatal period via spine radiography and confirmed by spine MRI, and autism spectrum disorder (ASD), diagnosed at two years of age. A careful clinical evaluation revealed only some mild facial dysmorphisms (i.e., protruding and crumpled ears) and a wide toe bilaterally.

WES was then performed and revealed the presence in the proband of the c.460C>T, p.(Arg154*) heterozygous nonsense variant in the *BMP2* gene (NM_001200.2). Pathogenic variants in *BMP2* are associated with autosomal dominant Short stature, facial dysmorphism, and skeletal anomalies with or without cardiac anomalies 1 (MIM: #617877) and the identified variant has already been reported as causative of this phenotype [24]. WES data analysis suggested that the variant was paternally inherited, but Sanger sequencing segregation did not confirm its presence in the father’s DNA. WES data was examined again with particular attention to the variant’s coverage in both the proband and the father: in the former, the alternative allele had a frequency of 50%, thus being compatible with the heterozygous state, whereas in the latter, the alternative allele had a frequency of 26%, thus prompting the suspicion of mosaicism. An allele-specific PCR was performed on the father’s DNA and allowed to detect both the wild-type allele and the mutated one, hence confirming the mosaic hypothesis.

In addition, a novel inframe deletion has been detected in the *INTS6L* gene (NM_182540.4). Variants in this gene have recently been described in two studies reporting patients affected by X-linked ASD and developmental disorder [25,26], thus designating *INTS6L* as a new candidate for this type of diseases. The identified variant (c.2552_2554delACA, p.(Asn851del)) is predicted as damaging by the in silico tool PaPI score and is not reported in any public database. Segregation analysis confirmed that the variant was maternally inherited and present at the hemizygous state in the proband. To our knowledge, Patient 5 is the third reported subject that presents a neurodevelopmental disorder and also carries a variant within the *INTS6L* gene. In this light, the identification of further patients together with the implementation of functional studies are mandatory to corroborate this genotype-phenotype correlation and confirm the specific role of this gene in neurodevelopmental disorders.

### 3.2. Patients Who Present Overlapping Phenotypes Due to the Contribution of Two Different Loci

#### 3.2.1. Patient 6: Drug-Resistant Epilepsy

Patient 6 (Figure 2F) is a two-years-old boy with a history of drug-resistant epilepsy, firstly appeared at two months of age. Video-EEG recording coupled to Electromyography (EMG) polygraphy recorded several nonfebrile ictal episodes, characterised by loss of consciousness, fixed stare, and variable motor manifestations, thus fostering the initiation of anticonvulsant therapy, that, however, resulted ineffective.

WES analysis of the trio revealed the presence in the proband of two variants in epilepsy-associated genes, *SCN1A* (NM_001202435.1) and *CSNK2B* (NM_001320.5). As regards *SCN1A*, the splice-site c.695-1G>A variant was identified at the heterozygous state; it affects a splice acceptor site, is predicted as damaging by the in silico tools DANN and dbscSNV scores, and has already been reported as possibly associated with Dravet syndrome (MIM: #607208) [27]. Conversely, variants in *CSNK2B* have recently been associated with the Poirier-Bienvenu neurodevelopmental syndrome (MIM: #618732), a neurological disorder characterised by early-onset and possibly refractory seizure, intellectual disability of various degree, and ASD. The c.384_394delAGGTGAAGCCA, p.(Pro128fs) frameshift variant was identified in the proband at the heterozygous state and has recently been reported as pathogenic in a small cohort of Poirier-Bienvenu patients [28]. Familial segregation analysis confirmed that both variants originated *de novo* in the proband.

#### 3.2.2. Patient 7: Epileptic Encephalopathy

Patient 7 (Figure 2G) is a four-year-old girl affected by epileptic encephalopathy. She has a positive family history, since her mother reported to suffer from tonic-clonic seizure from the age of 12 and one of her mother’s sisters presents epilepsy and intellectual disability.

The proband has experienced her first absence seizure at five months of age: brain MRI highlighted a thinning of the arcuate fasciculus that connects the Broca and Wernicke’s areas, and the EEG revealed the presence of interictal epileptiform discharges, thus fostering the diagnosis of epileptic encephalopathy. Furthermore, the girl also presented a complex neurodevelopmental disorder, characterised by ataxic gait, global developmental delay with severe speech impairment, ASD, and sleep disorder with frequent awakenings. A careful clinical evaluation revealed mild dysmorphisms, as a square face and small ears.

DNA of the proband’s father was not available since he passed away prior to the analysis, therefore WES was performed only on the girl’s DNA and identified two heterozygous variants in two different epilepsy-associated genes, *CACNB4* (NM_000726.5) and *ZEB2* (NM_014795.4). The missense variant in *CACNB4* (c.1418G>A, p.(Arg473His)) has already been reported as causative of Generalised idiopathic epilepsy (MIM: #607682) [29]. The novel missense variant in *ZEB2* (c.905G>A, p.(Arg302Gln)) affects a highly conserved residue, is predicted as damaging by all in silico tools employed in the analysis, is not present in the gnomAD database, and has been reported three times as Variant of Uncertain Significance (VUS) in ClinVar (RCV000717537, RCV001209725, RCV000159444). Additionally, a different variant affecting the same residue (c.904C>T, p.(Arg302*)) has already been reported in the literature in patients affected by Mowat-Wilson syndrome (MIM: #235730) [30] and epilepsy and neurodevelopmental disorders [31]. Segregation of both variants was performed in the proband’s mother and did not identify any of them.

## 4. Discussion

The introduction in the clinical setting of high-throughput sequencing technologies, such as WES, has enormously implemented our ability to achieve a precise molecular diagnosis even in the most complex cases. WES offers the possibility to simultaneously analyse all protein-coding genes, thus covering the majority of known pathogenic variants and providing a detailed analysis of the genetic makeup of a patient in a timely and cost-effective manner [32]. The huge amount of available data also opens up the opportunity to apply a genotype-first approach to identify a molecular diagnosis and this appears particularly useful in patients with entangled clinical pictures. Indeed, whenever a patient’s signs and symptoms do not fit into a known syndromic pattern, two different possibilities are generally considered: a phenotypic expansion, with more severe or new characteristics added to a well-recognised condition, or the identification of an apparently novel disease [33]. Syndromes present a multisystemic and multiorgan involvement, but not all patients affected by the same condition display identical characteristics and equal severity. As a consequence, a certain interindividual variability and a clinical heterogeneity are usually expected. In this light, whenever a patient displays the peculiar features of a known syndrome but also some additional phenotypic characteristics, geneticists usually consider the latter as an ancillary clinical manifestation of the specific disorder, thus identifying a phenotypic expansion [34]. On the other hand, whenever patients present an association of signs and symptoms that has never been reported before, the clinical suspicion of a novel genetic disorder arises. In these cases, WES grants the possibility to simultaneously analyse a huge amount of data and, as already discussed, possibly identify new disease-causing genes never implicated in Mendelian disorders before [35].

**Figure 3:**
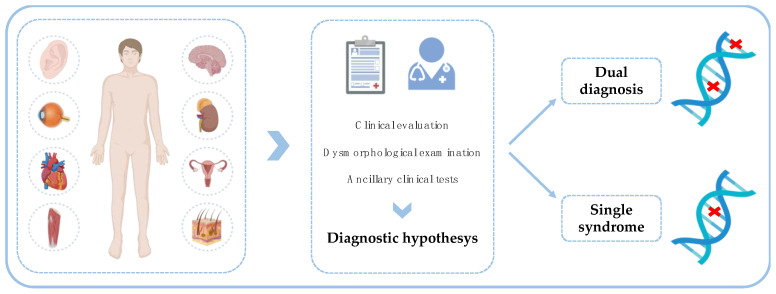
Diagnostic work-up of patients presenting complex clinical pictures. The first step consists in a detail clinical characterisation that is achieved through an anamnesis collection, a dysmorphological examination, and ancillary laboratory and imaging investigations. This leads to the formulation of a diagnostic hypothesis, that could immediately consider the possible presence of a dual molecular diagnosis or, on the contrary, contemplate the existence of a single syndromic condition.

In this context, the opportunity to screen the entire exome in a hypothesis-free manner has highlighted another compelling scenario: the presence of dual molecular diagnoses [2,3,33,36] (Figure 3). Although patients affected by multiple Mendelian disorders are being increasingly recognised and several case reports have recently described patients with multilocus genetic variation [6,8,9,11,37,38,39], this occurrence is still often overlooked. Indeed, there is a lack of comprehensive studies that systematically investigate the possible presence of pathogenic variants in multiple genes in large cohorts of unselected patients. In this light, our study underlines how a thorough and unbiased analysis of WES data could lead to the identification, especially in complex patients, of more than one genetic disorder, thus fostering geneticists to be aware of such occurrence and be prone to consider this hypothesis whenever examining a subject with atypical features.

Our study confirms that the identification of patients affected by dual molecular diagnoses is not a rare event: in our cohort they represent 5.1% of all molecularly solved cases, in line with reported literature data [2,3,4]. Our patients may be divided into two different groups: A) subjects presenting distinct phenotypes due to two different variants in two independently segregating loci; B) subjects presenting overlapping characteristics that may be explained by either of the two identified variants. Regarding the first category, it is worth noting that only one patient was suspected ab initio to be affected by two different diseases. Indeed, Patient 1 presented a Syndromic craniosynostosis that prompted a gestalt diagnosis of Apert syndrome. However, upon a detailed analysis of the clinical features associated with this condition, it emerged that her other peculiar sign, namely *xerosis cutis*, is not part of the well-established characteristics of *FGFR2*-associated craniosynostosis. This awareness suggested to further evaluate WES data to verify the possible presence of pathogenetic variants in ichthyosis-associated genes. Only coupling a detailed phenotypical characterisation with a deep analysis of sequencing data, it was possible to identify in this patient both molecular diagnoses. Concerning all other patients belonging to group A, the first clinical hypothesis was always a single-gene defect that could explain all signs and symptoms, considering that usually, in medicine, a single pathogenetic cause is more common than multiple ones [40]. This “parsimony principle” has indeed been challenged by Patients 2, 3, 4, and 5, whose clinical characteristics have eventually been explained by two different variants in two different genes, both responsible of distinct syndromes that were properly recognised only upon reverse-phenotyping.

As regards group B, it is interesting to notice how both patients presented with epilepsy, albeit of different types. Epilepsy is one of the most common neurological disorders and is characterised by a high clinical and genetic heterogeneity, with more than 900 genes associated with this phenotype [41]. In this light, if a patient is found to be a carrier of more than one variant in more than one epilepsy-associated gene, it could be extremely challenging to precisely define the role and the contribution of all identified variants in epileptogenesis [42,43]. As a consequence, in order to delve deeper into the possible underlying mechanisms, an in silico pathway analysis has been performed to assess protein-protein interactions. In Patient 6, affinity chromatography and sequencing-on-chip assays showed that the proteins encoded by both *SCN1A* and *CSNK2B* directly interact with the protein encoded by the *KMT2A* gene [44]. The latter is involved in the methylation of histone H3 lysine 4 (H3K4), which mediates chromatin remodelling. Variants in the *KMT2A* gene are associated with Wiedemann-Steiner syndrome (MIM: #605130), a neurodevelopmental disorder characterised by intellectual disability, hypertrichosis cubiti, short stature, and typical facial features. It is interesting to note that also patients affected by this condition may present epilepsy. As a consequence, it may be argued that all three genes belong to a common molecular pathway involved in epileptogenesis. Additionally, in Patient 7, affinity chromatography assay showed that the proteins encoded by both *CACNB4* and *ZEB2* directly interact with the protein encoded by the *TNIK* gene [45]. *TNIK* encodes a serine/threonine kinase that activates the Wnt signalling pathway. The canonical Wnt/β-catenin pathway is necessary for processes involved in early brain development and its dysregulation has been implicated in several neurological disorders, including epilepsy. It could therefore be hypothesised that both *CACNB4* and *ZEB2* may be involved in the same epileptogenic pathway through Wnt signalling alteration. Indeed, the activation of the Wnt/β-catenin pathway has been associated to seizure-induced changes in the brain, as hippocampal neurogenesis, apoptosis pathway activation, hippocampal sclerosis, and mossy fibers sprouting [46]. However, further functional in vitro and in vivo studies are mandatory, both to verify the pathogenicity of previously unreported variants (e.g., the novel missense variant in *ZEB2* identified in Patient 7) and to verify the exact molecular mechanisms underlying these predicted interactions towards the determination of the epileptic phenotype.

An additional interesting consideration concerns the inheritance patterns of multilocus variations in our patients. In line with literature data [2], pathogenic or likely pathogenic variants in autosomal dominant (AD) disease genes were the most common, being identified in ten out of 14 diagnoses (71.43%). Among them, four variants where inherited from a parent (40%), including the peculiar case of Patient 5, whose father was a mosaic carrier of a known pathogenic variant in the *BMP2* gene and did not display any associated clinical features. Additionally, four variants in AD genes (40%) had a *de novo* origin in the proband, supporting the hypothesis that private variants are a major cause of genetic disorders [47]. Finally, for two variants in genes associated with AD disorders (20%), it was not possible to determine the pattern of inheritance: they were both identified in the same proband, whose father’s DNA was not available for familial segregation analysis. In three out of 14 cases (21.43%), a disease-causing variant has been identified in autosomal recessive disease genes: in one case two compound heterozygous variants were detected (Patient 1) whereas in two cases homozygous variants were recognised (Patients 2 and 3). In the last two families, consanguinity was not overtly reported but, in both cases, the proband’s parents declared to come from the same small town. Finally, only one diagnosis (7.14%) could be ascribed to an X-linked disease gene (Patient 5), highlighting the relevance of maternally inherited X-linked variants in the etiopathogenesis of Neurodevelopmental disorders [26].

Overall, as it indeed appears from the cases reported in this study, the presence of dual molecular diagnoses represents a challenge to the clinician, since complex phenotypes might be misleading and drive to a wrong interpretation. In this light, only a detailed analysis of patients’ clinical features together with a careful examination of WES data can provide the correct molecular diagnosis. It is therefore mandatory to analyse sequencing data within a multidisciplinary team that gathers all healthcare professionals involved in the clinical management, as geneticists, cardiologists, neurologists, otorhinolaryngologists, ophthalmologists, and radiologists. Delivering integrated care is also fundamental to establish the most appropriate follow-up and treatment, especially in neonatal and paediatric patients, since the presence of multiple molecular diagnoses might have an impact on medication response and interactions, [7]. Lastly, the presence of more than one genetic condition demands a careful examination of all at-risk family members to offer appropriate counselling and precisely define familial recurrence risk, thus guaranteeing an informed and responsible family planning to all involved subjects.

## 5. Conclusions

In conclusion, our study underlines the importance of challenging the classical concept of “one explanation covers them all” whenever trying to identify the molecular defect underlying complex phenotypical presentations. Dual molecular diagnoses represent a fascinating model of complex inheritance and should always be suspected when patients present atypical clinical features, thus fostering a deeper and more detailed analysis of sequencing data. This is because the identification of a correct molecular diagnosis is the indisputable starting point for a truly personalised and safe clinical management.

## Figures and Tables

**Table 1:** List of dual molecular diagnoses identified through WES. Patient: Patients 1, 2, 3, 4 and 5 belong to group A (patients who present distinct phenotypes, due to each of the two different underlying genetic diseases), whereas Patients 6 and 7 belong to group B (patients with overlapping clinical features that may be underpinned by both the identified genetic variations). Clinical features: main characteristics displayed by each patient. Molecular diagnosis: the details of both genetic diagnoses are reported for every patient as follows: Gene: list of genes carrying the identified variants with NCBI RefSeq accession number of the considered protein-coding transcripts (NM_). cDNA change and Protein change: variant description according to the Human genome Variation Society (HGVS) nomenclature guidelines. Genotype: homozygous: the same variant is present on both alleles; heterozygous: the variant affects only one allele; compound heterozygous: two different variants are present on each allele; hemizygous: the variant affects a gene located on the X chromosome in a male subject. Inheritance: inheritance pattern of every identified variant established after parental segregation. ACMG/AMP classification: variants pathogenicity according to ACMG/AMP guidelines. References: PubMed unique IDentifier of publications reporting each variant. *: stop codon. NA: Not Available.

Patient	Clinical Features	Molecular Diagnosis	Gene	cDNA Change	Protein Change	Genotype	Inheritance	ACMG/AMP Classification	References
1	Syndromic craniosynostosis and Xerosis cutis	First	*FGFR2* (NM_000141.4)	c.940G>T	p.(Ala314Ser)	Heterozygous	*de novo*	Likely pathogenic	PMID: 9521581
		Second	*FLG* (NM_002016.1)	c.7339C>T c.3191G>A	p.(Arg2447*) p.(Trp1064*)	Compound heterozygous	Paternal Maternal	Pathogenic Uncertain significance	PMID: 17417636 PMID: 32018027
2	Drug-resistant epilepsy and Congenital heart defect	First	*SCN1A* (NM_001165963.1)	c.2591_2593 delTGC	p.(Leu864del)	Heterozygous	*de novo*	Pathogenic	NA
		Second	*MMP21* (NM_147191.1)	c.903G>A	p.(Met301Ile)	Homozygous	Paternal and Maternal	Uncertain significance	NA
3	Intellectual disability and Hypertrophic cardiomyopathy	First	*ELP2* (NM_001242875.1)	c.1580G>A	p.(Arg527Gln)	Homozygous	Paternal and Maternal	Likely pathogenic	PMID: 33510603
		Second	*MYBPC3* (NM_000256.3)	c.913_914 delTT	p.(Phe305Pro fs*27)	Heterozygous	Paternal	Pathogenic	PMID: 18533079
4	Syndromic intellectual disability	First	*ZMIZI* (NM_020338.3)	c.1984A>G	p.(Asn662Asp)	Heterozygous	Maternal	Uncertain significance	NA
		Second	*DSG2* (NM_001943.3)	c.621_626 delTCCTCC	p.(Tyr207_Pro209 delinsTer)	Heterozygous	Maternal	Pathogenic	NA
5	Multiple malformations and Autism spectrum disorder	First	*BMP2* (NM_001200.2)	c.460C>T	p.(Arg154*)	Heterozygous	Paternal mosaicism	Pathogenic	PMID: 29198724
		Second	*INTS6L* (NM_182540.4)	c.2552_2554 delACA	p.(Asn851del)	Hemizygous	Maternal	Uncertain significance	NA
6	Drug resistantepilepsy	First	*SCN1A* (NM_001202435.1)	c.695-1G>A	NA	Heterozygous	*de novo*	Pathogenic	PMID: 28441824
		Second	*CSNK2B* (NM_001320.5)	c.384_394del AGGTGAAGCCA	p.(Pro128fs)	Heterozygous	*de novo*	Pathogenic	PMID: 35205321
7	Epileptic encephalopathy	First	*CACNB4* (NM_000726.5)	c.1418G>A	p.(Arg473His)	Heterozygous	NA	Uncertain significance	PMID: 33144682
		Second	*ZEB2* (NM_014795.4)	c.905G>A	p.(Arg302Gln)	Heterozygous	NA	Likely pathogenic	NA

## Data Availability

Data presented in this study are available upon request to the corresponding author. Data are not publicly available due to privacy restriction.

## Supplementary Materials

The following supporting information can be downloaded at: https://www.mdpi.com/article/10.3390/genes13112023/s1, Figure S1: Bioinformatic pipeline for the selection of the final list of variants; Table S1: Coverage statistics; Table S2: Variant calling and filtering.
