# Supplementary material for: Challenging Occam’s Razor: Dual Molecular Diagnoses Explain Entangled Clinical Pictures
Source: Genes (Basel). 2022 Nov 3;13(11):2023. doi: 10.3390/genes13112023 (PMC9690221; doi:10.3390/genes13112023)
Supplement: Supplementary file 1 [file genes-13-02023-s001.zip › Table_S1.pdf]

| Samples | Target Region Average Coverage | Target Region Minimum Coverage | Target Region Maximum Coverage | Number of Poorly Covered Regions | Number of Poorly Covered Exons |
|---|---|---|---|---|---|
| All samples | 70 | 18 | 4784 | 118858 | 27746 |
| Patient 1 | 69 | 37 | 4604 | 120424 | 15806 |
| Patient 2 | 76 | 34 | 5097 | 118485 | 48557 |
| Patient 3 | 70 | 1 | 5196 | 119817 | 15677 |
| Patient 4 | 79 | 32 | 5545 | 117523 | 48851 |
| Patient 5 | 76 | 5 | 5754 | 118301 | 48068 |
| Patient 6 | 70 | 7 | 5794 | 118664 | 49305 |
| Patient 7 | 71 | 33 | 4529 | 120018 | 16990 |

**Table S1. Coverage statistics.** Average coverage statistics of all 342 sequence samples and detailed coverage statistics of Patients 1 to 7 are reported. Minimum coverage is reported considering bases with coverage depth >15. A coverage <15 is considered to define the number of poorly covered regions and poorly covered exons (target regions).
