# Supplementary material for: Challenging Occam’s Razor: Dual Molecular Diagnoses Explain Entangled Clinical Pictures
Source: Genes (Basel). 2022 Nov 3;13(11):2023. doi: 10.3390/genes13112023 (PMC9690221; doi:10.3390/genes13112023)
Supplement: Supplementary file 1 [file genes-13-02023-s001.zip › Table_S2.pdf]

Samples	Total number of variants	On-target variants	Off-target variants	Variants reported in dbSNP	Variants with MAF<0.1%	Coding and splicing variants	Pathogenic, likely pathogenic and VUS	Pathogenic and likely pathogenic in ClinVar	Familial pattern of inheritance
Patient 1	130949	27583	20565	24220	3195	722	652	2	9
Patient 2	126897	26438	19689	23582	2290	521	466	2	8
Patient 3	122810	27033	20016	24117	2156	521	471	4	6
Patient 4	128512	27097	20053	24094	2110	492	450	4	6
Patient 5	128719	26708	19899	23806	2246	493	442	5	5
Patient 6	128748	27441	20609	23962	3231	743	660	5	19
Patient 7	125625	26755	19893	23678	2033	513	448	1	16

Table S2. Variant calling and filtering. For each sample, the total number of called variants are reported, divided in on-target and off-target calls. On-target variants were checked in NCBI dbSNP build 155 to verify whether they have been previously reported. Variants were therefore analysed through the enGenome Expert Variant Interpreter (eVai) software, according to the following criteria: MAF (Minor Allele Frequency) <0.1%, effect (only coding and splicing variants were considered), and pathogenicity (evaluated with the ACMG/AMP guidelines and through *in silico* prediction tools – only pathogenic, likely pathogenic and Variants of Uncertain Significance (VUS) were considered). The retained variants were both checked in ClinVar, to verify whether they had already been reported as pathogenic or likely pathogenic, and analysed according to each patient's relevant familial pattern of inheritance (autosomal dominant, autosomal recessive, X-linked dominant, X-linked recessive, Y-linked, and *de novo*). Variants were then discussed within a multidisciplinary team and only variants of interest were confirmed by Sanger sequencing.
