# Supplementary figures and images for: Challenging Occam’s Razor: Dual Molecular Diagnoses Explain Entangled Clinical Pictures
Source: Genes (Basel). 2022 Nov 3;13(11):2023. doi: 10.3390/genes13112023 (PMC9690221; doi:10.3390/genes13112023)

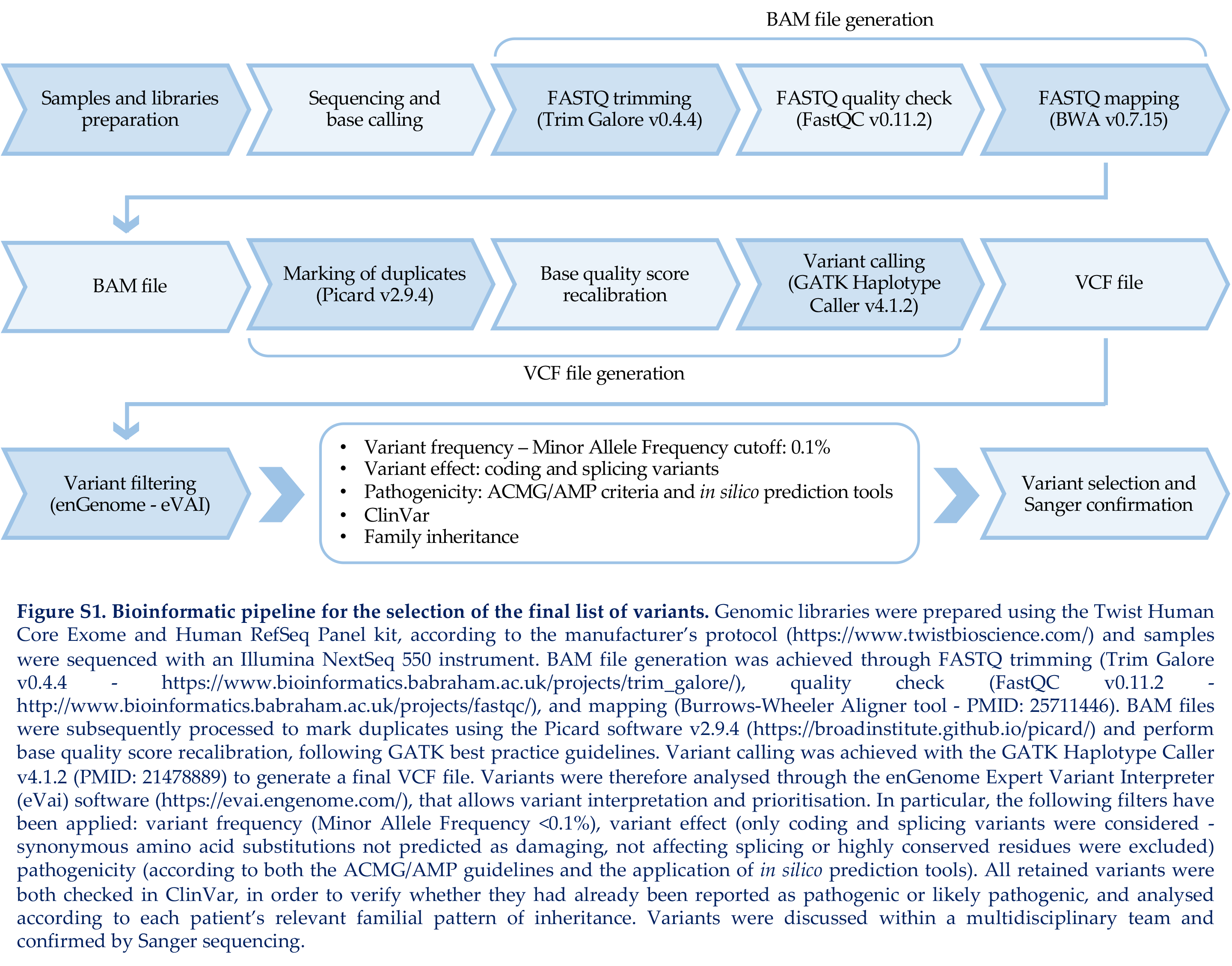

Supplement: Supplementary file 1 [file genes-13-02023-s001.zip › Figure S1_300dpi.tif]
